# p73 Alternative Splicing: Exploring a Biological Role for the C-Terminal Isoforms

**DOI:** 10.1016/j.jmb.2018.04.034

**Published:** 2018-06-22

**Authors:** Polina Vikhreva, Gerry Melino, Ivano Amelio

**Affiliations:** 1 MRC Toxicology Unit, University of Cambridge, United Kingdom; 2 Department of Experimental Medicine and Surgery, IDI-IRCCS, University of Rome Tor Vergata, Italy

**Keywords:** neurodevelopment, splicing isoforms, transcription, p53 family, p73, SAM, sterile alpha motif, YAP, Yes-associated protein, SUMO, small ubiquitin-like modifier, RACK1, receptor for activated C kinase-1

## Abstract

p73 (encoded by *TP73* gene) is a p53 related protein that functions as a transcriptional factor. Similarly to p53, following DNA damage, p73 is stabilized and activated and controls expression of target genes that are involved in the regulation of cycle arrest and apoptosis. However, great complexity to the function of this gene is given by the wide range of its non-tumor-related roles, which include neurological development, ciliogenesis and fertility. From the structural point of view, p73 displays an intricate range of regulations because it can be expressed both as an N-terminally deleted dominant-negative isoforms and as multiple alternatively spliced C-terminal isoforms, which can include or not a sterile alpha motif domain. More is known about the functions of the N-terminal isoforms of p73 (TAp73 and ΔNp73) and their opposing pro- and anti-apoptotic roles, whereas the functional differences of the distinct C-terminal splice forms of p73 are very far away from been defined. Here we summarize the current available literature regarding p73 C-terminal isoforms and the contribution of the sterile alpha motif domain to p73 function, trying to provide an unified view in this complex and sometime controversial field. Current data indicate that the full-length, TAp73α, is the major, if not the exclusive, isoform detected in physiological systems, indicating that detailed spatio-temporal expression analysis and functional studies are highly demanded to support a physiological role for the p73 alternative splicing. With this article, we also aim to emphasize the need to further investigation on the topic, refocusing the attention on what we believe are the most relevant unanswered questions.

## Highlights

• p73 is a p53 family member involved in tumor suppression and development.
• Alternative splicing and dual promoter usage generate a wide range of p73 isoforms.
• Alternative splicing at the C-terminal region of p73 generates at least seven different splice forms.
• The current data indicate that the full-length p73 isoform, p73α, is the major expressed form and is required for p73 function.

## Graphical Abstract

**Figure:**
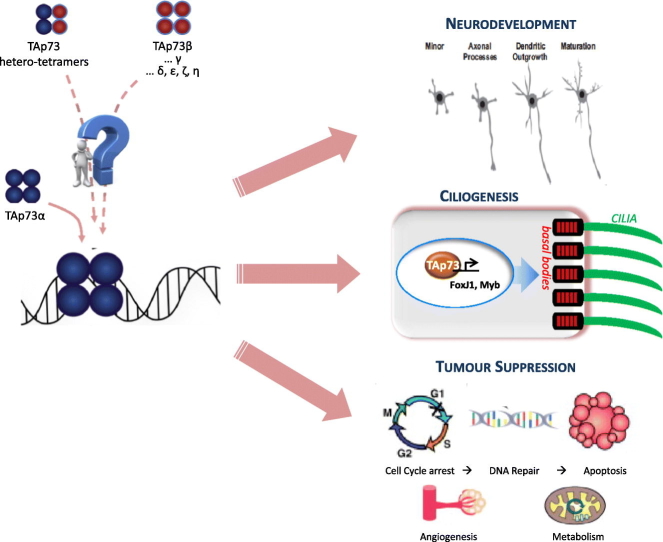

## p73: a p53 family member

Greater than 30 years have passed since the discovery of p53, and it still remains one of the most studied proteins in cancer fields and one of the most powerful tumor suppressors. Given its crucial function, p53 was suitably named the “guardian of the genome”. Two p53-related genes *p73* and *p63* were discovered approximately 20 years ago [1], [2], giving rise to the *p53 family*.

A major functional similarity of this family of transcriptional factors is the ability to execute the cellular response to genotoxic stress. Upon DNA damage, following slightly different mechanisms, p53, p63 and p73 are stabilized and activated, and promote regulation of a shared transcriptional signature that results in cell cycle arrest, attempt to repair DNA and, if needed, eventually apoptosis [3], [4]. The fact that this signaling is common to all the family members is suggestive that this might be the evolutionary most ancient function. The oldest family member, p63 indeed made its appearence in evolution to preserve genome stability of female germ cells, a role that p63 still kept in mammals and that probably evolved in the tumor suppression function of p53, when more complex organisms required preservation of the somatic cells genome to prevent cancer [5]. However, p63 and p73 proteins each have very distinct and specific DNA damage-independent roles: p63 is a master regulator of epidermal development and homeostasis [6], [7], [8], [9], whereas, as detailed in the following sections of this review, the p73-knockout has revealed an unexpected role for p73 in development of the nervous system [10], [11] and ciliogenesis [12], [13], [14].

From the structural point of view, the family members are also very similar. However, the presence of sterile alpha motif (SAM) at the C-terminal of p73 and p63 proteins increases the structural complexity of these members and might account for the unique signaling network of regulators and transcriptional targets [4]. All the family members are expressed as N-terminally containing (TA) or deleted (ΔN) isoforms and multiple alternative splicings at the 3′ region of the pre-RNA give rise to C-terminal isoforms, which for p63 and p73 can contain or exclude the SAM domain. The C-terminal of p63 has been widely studied in physiological and pathological contexts. Heterozygous mutations in *TP63 gene* are causative of a group of autosomal dominant human conditions, associated with combinations of ectodermal dysplasia, orofacial clefting and limb malformations [15]. A particular subgroup of these, the ankyloblepharon-ectodermal defects-cleft lip/palate syndrome, correlates with mutations clustered in the C-terminal region of p63 protein, generally involving amino acid substitutions in SAM domain or TI domain [15]. Deletion of the p63 C-terminus in mice also leads to ectodermal malformation and hypoplasia, accompanied by a reduced proliferative capacity of epidermal progenitor cells [16]. The study of p63 SAM domain has also led to identification of potential key interactions, including lipids [17], but the full mechanistic details of its contribution to p63 functions are still under investigation. The 3′ region of p73 is even more complicated as it can undergo a higher number of alterative splicing, producing at least seven splice forms (α, β, γ, δ, ε, ζ, η), whereas p53 and p63 can produce only 2 or 3 isoforms (α, β, γ) [4].

The focus of this article is to review all the literature regarding the function of the wide range of p73 C-terminal isoforms. More information is available about the functions of the N-terminal isoforms of p73 (TAp73 and ΔNp73) and their opposing pro- and anti-apoptotic roles. Distinct contributions of the different p73 C-terminal isoforms to the p73 function are indeed unclear. However, this information is important to understand the potential role of each isoform to development, oncogenesis and other human pathologies. This article will try to point attention of relevant scientific community on the need of further understanding of the C-terminal isoforms of p73, summarizing the available literature and revising old findings in the light of more recent progress. We also aim to set priorities for future studies on the topic.

## Structure of *p73* gene

**Fig. 1:**
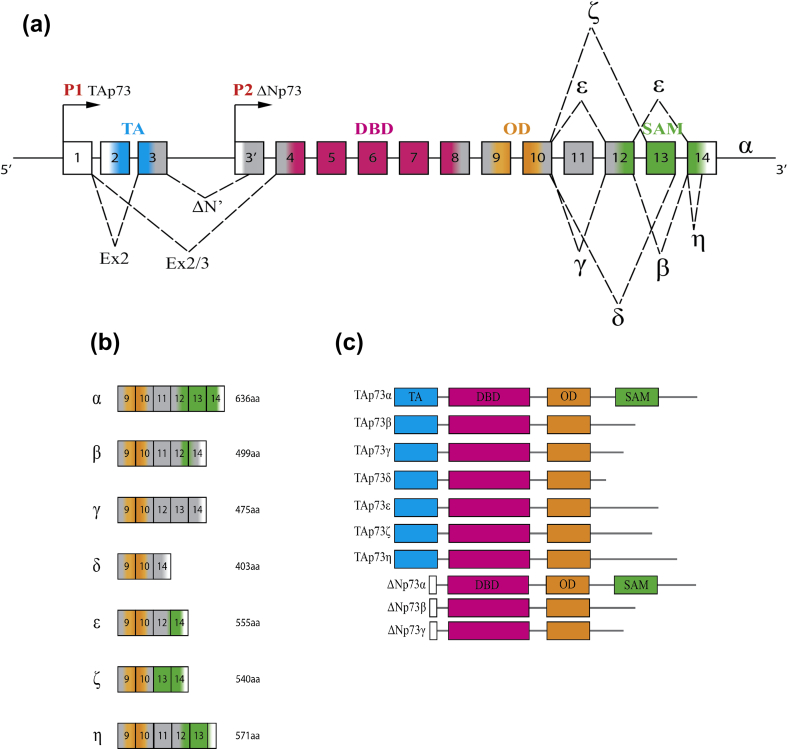
A schematic representation of the human *TP73* gene structure and protein domains. (a) The *TP73* gene consists of 15 exons (white, untranslated region). Several p73 isoforms are expressed due to the usage of alternative promoters (P1 and P2 arrows, isoforms TAp73 and ΔNp73) and splicing sites (dotted lines, N-terminal isoforms: ΔEx2, ΔEx2/3, ΔN′; C-terminal isoforms: α, β, γ, δ, ε, ζ, η). TA, transactivation domain; DBD, DNA-binding domain; OD, oligomerization domain; SAM domain, sterile alpha motif domain. (b) Schematic representation of genomic organization of C-terminal p73 isoforms (α, β, γ, δ, ε, ζ, η). (C) Some possible p73 protein isoforms: TAp73α, TAp73β, TAp73γ, TAp73δ, TAp73ε, TAp73ζ, TAp73η, ΔNp73α, ΔNp73β and ΔNp73γ isoforms. TA, transactivation domain; DBD, DNA-binding domain; OD, oligomerization domain; SAM domain, sterile alpha motif domain.

The *TP73* (*Tumor P*rotein p*73*) gene consists of 15 exons and is located on the short arm of chromosome 1 (1p36.33) [1]. The *TP73* gene gives rise to different mRNAs, which are translated into several different proteins. Various mRNAs arise due to the use of two alternative promoters, namely P1 in the 5’UTR upstream of exon 1 and P2 located between exons 3 and 4, and the alternative splicing of the N-terminal and C-terminal (Fig. 1a–c). Transcription from two different promoters on the *TP73* gene results in the generation of TAp73 and ΔNp73 (Fig. 1a). The former contains a transactivation (TA) domain, which is encoded by exons 2 and 3. In contrast, ΔNp73 is shorter and does not contain TA domain. The other group of N-terminal truncated p73 isoforms arises from alternative splicing of the N-terminus of the transcript generated from the P1 promoter (ΔEx2p73, ΔEx2/3p73 and ΔN′p73) [18].

Isoform-specific knockout mouse models for the N-terminal isoforms demonstrated that TAp73 depletion facilitates cancer development and leads to important developmental issues, whereas depletion of ΔNp73 reduces tumor growth [10], [19]. Expression of a TAp73 is also important in neurogenesis and pheromonal signaling and for normal dynamics of cerebral fluid [11]. Evidence suggests that TAp73 is required also for proper differentiation of multiciliated cells by directly regulating transcription factor Forkhead box J1 (Foxj1), which is required for the transactivation of genes encoding proteins involved in ciliogenesis [13]. p73-null and TAp73-null mice display defective ciliogenesis in upper respiratory airways, associated with impaired clearance of inhaled pollutants and pathogens from airways [14].

Many splicing events occur at the 3′ end, in the part of the sequence that is not found in the *p53* gene. At least seven different 3′ terminal transcripts are known (α, β, γ, δ, ε, ζ, η) to be generated at the C-terminus and express only one of two N terminal sequences (TA or ΔN) [20] (Fig. 1a). Collectively, the *TP73* gene expresses at least 29 different transcripts, but it remains uncertain how many of these transcripts are truly expressed as proteins (some are shown in Fig. 1b, c). Carboxy-terminal isoforms are due to alternative splicing of exons 11, 12 and 13, which code for the SAM domain. Given that the *p53* gene lacks the SAM domain, it is likely that the SAM domain is important for the differential activities of p73. p73α is the only isoform that contains a fully functional SAM domain. The p73β, p73γ, p73δ and p73η isoforms are truncated forms of full-length p73α, so the alternative reading frame that is created by splicing events generates a STOP codon. The p73α transcript includes exons 1–14, whereas the p73β transcript lacks exon 13. The p73γ isoform contains all the exons coding for the SAM domain, but the splicing event at exon 11 creates a long alternative reading frame that leads to a shift of the reading frame formed from a different carboxyl terminus by a premature STOP codon [21]. The p73δ isoform lacks exons 11–13 and thus most of the carboxy-terminal region. Because of this feature, it is more similar to p53 than other p73 isoforms. The p73ε isoform has a carboxyl-terminal region that is composed of parts of the p73γ and p73α reading frames (it lacks exons 11 and 13). The p73ζ isoform has an internal deletion of exons 11 and 12. Therefore, it contains most of the SAM domain, although it lacks several residues that seem to be fundamental for stability and functionality. p73η is closely related to p73α but differs at exon 14.

## p73 SAM domain and transcriptional activity

p73α is the only isoform that contains a fully functional SAM domain. This has been described as a possible protein–protein interaction domain and might contribute to the control of p73 transcriptional activity [22], [23]. NMR spectroscopy resolved the three-dimensional structure of this domain from human p73. Residues 487 ± 554 form a 5-helix bundle with recapitulate structures of Ephrin receptor tyrosine kinases SAM domains, whose involvement in protein–protein interaction is well characterized and associated with regulation of the transcription [23], [24]. This structural similarity supports the arguments that p73 SAM domain is responsible for interactions involved in regulation of p73 transcriptional capability. However, the pattern of surface residues of p73 (spanning amino acids 487 ± 554) appears unrelated to that of the Eph receptors and mostly recapitulates p63 SAM domain surface. This might indicate that p63/p73 C-terminal regions could represent an independent unique class of protein domains structurally related to the SAM.

Experimental data indicate that the SAM domain influences the transcriptional activity of p73. C-terminally truncated p73 isoforms exhibit different ranges of transcriptional activities. Among them, the p73β isoform more actively induces apoptosis and is the strongest transcriptional activator, at least among characterized p53-target genes [25]. The p73γ isoform exhibits high expression in testis compared with other tissues and is a relatively weak transactivator of p53-target genes [21], [26]. Compared with other C-terminal isoforms, the p73η isoform is exclusively detected in lymph nodes [27]. This isoform exhibits considerable potential to transactivate p53-target genes and is comparable to p73α and p73γ. More similar to p73α, p73δ exhibits intermediate strength in transactivation [21], [26]. The functional role of the p73α C-terminal region was also investigated with a series of p73α truncation mutants. p73α(1–427) (lacking the most COOH-terminal region including a SAM domain) and p73α(1–548) (deleting an extreme COOH-terminal region except a SAM domain) displayed significant transcriptional ability on a luciferase-based reporter assay and higher DNA binding compared to the full-length p73α [28]. Overall these data confirmed the potential of the p73 C-terminal region for regulation of p73 activity.

The tetrameric state of p53, p63 and p73 has been considered one of the hallmarks of this protein family. p73 can form hetero-interactions with p63 and with a lesser extent aggregate with forms of mutant p53 [21], [29], [30]. Remarkably, due to structural differences, p73 cannot hetero tetramerize with wt p53. Structural studies on the C-terminal of the ancestral p53 family members, the *Caenorhabditis elegans* and *Drosophila* forms, CEP-1 and Dmp53, indicate a potential contribution of the SAM domain present in these proteins in the oligomerization of transcriptional factor. These additional structural elements were necessary for the integrity of the oligomerization domain of Dmp53 and CEP-1 proteins [31]. Remarkably, these are not present in human p53, but make these ancestral forms structurally closer to p63 and p73. These analyses indicate a contribution of the SAM domain in the stability of p73 tetramer and therefore in its transcriptional ability. Although there are no data formally proving a contribution of the SAM domain in p73 oligomerization, these findings are suggestive of a potential function of this domain in vertebrate p53 family members.

Tissue-specific expression of p73 C-terminal isoforms in mice indicates that although RNA of the C-terminal variants can be detected virtually in all organs, their expression is below the threshold for detection of the corresponding protein; p73α was indeed the only significantly expressed C-terminal isoform [32]. These analysis leads us to speculate that SAM domain is required for p73 developmental functions; however, this assumption requires validation in appropriate animal models.

## Roles of p73 isoforms in cancer

Mutations in *TP73* gene in cancer are very rare and *TP73*-deficient mice lack a spontaneous tumor phenotype [11]. However, isoform-specific knockout mice revealed that the depletion of TAp73 predisposes to cancer, whereas the absence of ΔNp73 decreases tumor growth [10], [19]. The relative expression of TAp73 and ΔNp73 isoforms is indeed probably responsible for the biological outcome of p73 expression. As a result, most studies in cancer-related fields focus their attention on the analysis of changes in TAp73 and ΔNp73 expression levels. Apoptotic activities of transcriptionally active p73 isoforms (TA) are commonly inhibited by dominant negative p73 isoforms (ΔN), which can counteract TAp73 tetramerization or compete for promoter binding. As a result, the relative ratio of different p73 isoforms is crucial for the cellular response to a chemotherapeutic agent [10], [33].

Tumors and cell lines with highly expressing p73 tend to display a complex profile of up to six different C-terminal splice variants, where p73α is still the major form, whereas normal and breast cancer tissues with low p73 mRNA levels exclusively express TAp73α. However, generally the low-expressing tissues display predominantly p73α, whereas overexpressing tumors show a complex pattern [34]. This is again suggestive that expression of alpha isoform is the major outcome of TP73 gene expression and the relevance and contribution of the other isoforms remain unclear.

The study of p73 C-terminal function in tumorigenesis is limited to *in vitro* studies largely based on overexpression approaches. The two mostly studied C-terminal isoforms are TAp73α and TAp73β. Overexpression of TAp73α, TAp73β and TAp73γ induces apoptosis in SaOs-2 cells by promoting transcriptional regulation of PUMA and Bax and mitochondrial translocation of Bax. Interestingly, transactivation capability on the pro-apoptotic factors and the extent of the apoptotic effect were largely higher in the shortest isoforms TAp73β and TAp73γ [3]. The p73-dependent pro-apoptotic programme was also associated with induction of the Cip/Kip family of cyclin-dependent kinase (CDK) inhibitor, p57^Kip2^. p57^Kip2^ appeared to be a selective transcriptional target of p73β isoform, responsible of its programmed cell death induction [35]. However, the biological relevance of these findings should be revised after re-analysis of the question with more modern technologies, as these results might be the consequence of overexpression at non-physiological level of short isoforms resembling p53 structure. More recent studies indicate that TAp73 contributes to genomic stability of somatic and germline cells. The interaction of TAp73α with kinetochore proteins Bub1 and Bub3 alters the mitotic checkpoints and deregulation of TAp73 in cancer might therefore have an impact on polyploidy [36], [37]. TAp73 was also shown to bind and regulate the hypoxia-inducible factor 1α (HIF-1α). In hypoxic tumor, expression of TAp73 represses activation of HIF-1, thus limiting tumor angiogenesis and therefore progression [30], [38], [39]. Additional contribution of p73 to tumor cell biology might be mediated by its effect on the cellular metabolism, which largely appears to support anti-oxidant defence and anabolic processes. TAp73 expression is associated with an increased serine/glycine synthesis, production of NADPH and GSH in different cancer models, and glutaminase-2 (GLS-2) and glucose-6-phosphate-dehydrogenase (G6PD) metabolic enzymes have been described as direct TAP73 targets [40], [41], [42], [43]. However, a detailed analysis of the differential effects of the C-terminal isoforms was not conduced.

**Fig. 2:**
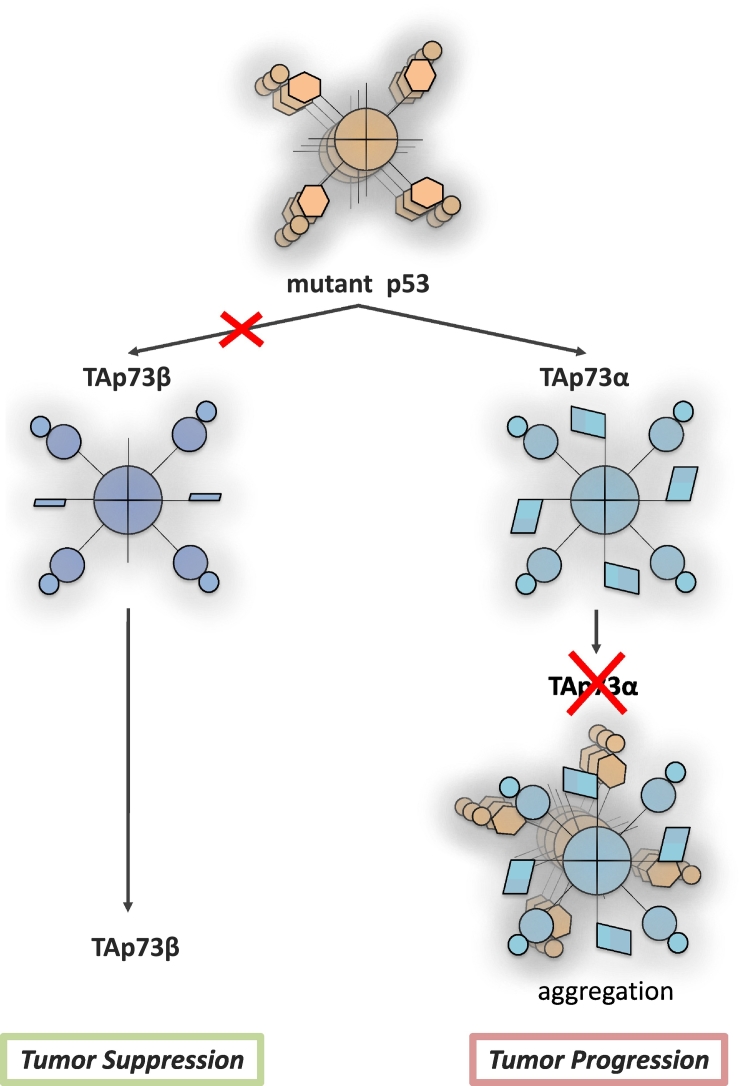
Aggregation propensity of p73 C-terminal isoforms with p53 mutants. The cancer hotspot mutation p53 R175H interacts with exclusively with the C-terminal of p73α, which includes SAM and TI domains. Amino acids corresponding to the TI domain of p73α are required for p53 R175H aggregation. Consequentially, the p73β isoform, which lacks the SAM and TI domains, is not susceptible to the inhibition mediated by p53 R175H aggregation. This suggests that p73β might preserve its tumor suppression ability in a p53 mutant genetic background.

Interesting insights into the contribution of the C-terminal region to a potential p73-related cancer phenotype have been provided by structural studies on the aggregation propensity of mutant p53 R175H on p73 C-terminal isoforms. Mutations in p53 are found in more than 50% of all the human cancers, and their oncogenic potential is associated with not only the loss of function of wt p53 but also expression of neomorphic forms of the protein originated by missense mutations. p53 conformational mutants, such as R175H, have been suggested to directly bind to p73 *via* a co-aggregation mechanism mediated by their unstructured DNA binding domains. This effect was postulated to repress any p73-dependent tumor suppression capability, resulting in more aggressive phenotype [44], [45]. Detailed mapping of the interaction motif of the region of p73α identified the last few amino acids of the protein, corresponding to a predicted transactivation Inhibitor domain, to mediate interaction with p53R175H. Consequentially, shorter C-terminal isoforms, such as TAp73β, appear insensitive to p53R175H, thus potentially conserving their tumor suppression properties in p53 mutant genetic background (Fig. 2) [46]. It still remains elusive whether a tumor can express TAp73β at sufficient levels and what are the underlining molecular mechanism forcing the shift from alpha to beta.

## Regulation of p73 C-terminal isoforms by selective post-translation modifications

**Fig. 3:**
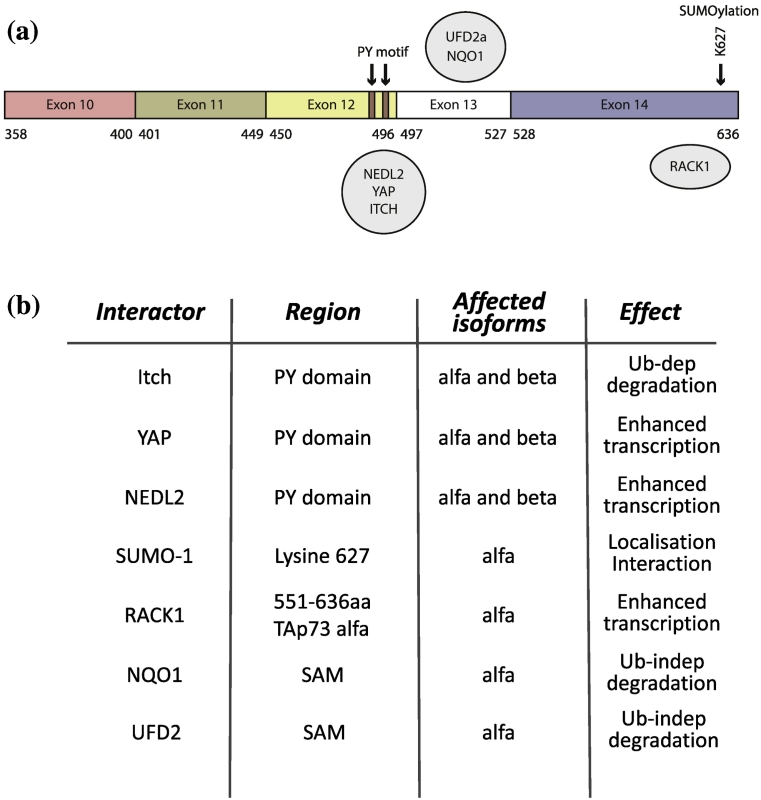
Interactors of p73α C-terminus. (A) Schematic representation of p73α C-terminus a corresponding interactors or posttranslational modifications. SUMOylation of p73 occurs at lysine 627 (K627). NEDL2, YAP and Itch share the region of interaction corresponding to the proline rich (PY) motif. NQO1 was described to interact with the SAM domain. RACK 1 interacts with the last few C-terminal amino acids of p73α.

Stabilization and activation of p53 follows a deeply studied pattern of post-translational modifications involving phosphorylation, acetylation, sumoylation and ubiquitination, with a major regulation step represented by the interaction with ubiquitin-ligase MDM2, which controls its protesomal degradation [47]. p73 does bind MDM2, but in contrast with p53, this interaction does not mediate a control of the stability; however, stabilization of p73 following DNA damage and other stress appears to be the predominant mechanism of regulation for its expression [4]. The U-box-type E3/E4 ubiquitin ligase UFD2a was functionally associated with p73α, and in particular, the SAM domain appears to be involved in a physical interaction. UFD2a indeed mediates the proteasomal turnover of p73 in a mechanism that does not involve ubiquitination [48]. The Hect ubiquitin-ligase, Itch, was identified as an exclusive binding partner of p73. Upon DNA damage, Itch is downregulated leading to an increased level of p73 [49]. However, WW domain of Itch recognizes p73 on it proline-rich (PY) domain, which is encoded by exon 12. As a result, Itch has no effect on the shortest p73 proteins, such as the γ and δ isoforms, and for the same reason does not affect p53 stability [49]. So far, Itch is the major recognized regulator of p73 stability (Fig. 3a, b).

The PY domain of p73 is involved also in other potentially key protein interactions. The PY domain indeed interacts with the WW domain of with the Yes-associated protein (YAP). The motif binds to a 40-amino-acid structural domain known as the WW domain that is organized to form a three-stranded, antiparallel β sheet containing two tryptophan (W) residues spaced 20–22 amino acids apart [50]. YAP enhances p73α-dependent transcriptional activation in p53-deficient H1299 cells. YAP binds p73α and p73β, but it does not interact with p73γ and other shorter isoforms. In addition to this, NEDD4-related E3 ubiquitin ligase NEDL2 can also form a complex with the C-terminus of p73α and p73β isoforms [51]. NEDL2 overexpression resulted in the ubiquitination of p73, which leads to NEDL2-mediated stabilization of p73 and enhanced p73-dependent transcriptional activation. The C-terminal SAM domain region of p73α is also required for direct binding to NAD(P)H quinone oxidoreductase 1 (NQO1), which leads to it ubiquitin-independent proteasomal degradation [52].

Activation and stabilization of p73 is associated with other post-translational involving the C-termini. The C-terminal lysine 627 can be covalently modified by SUMOylation (small ubiquitin-like modifier, or SUMO). Lysine 627 is contained in the C-terminal region of the p73α isoform not p73β, suggesting another distinct regulation for the individual isoforms (Fig. 3a). SUMOylation of p73α is not required for p73 degradation, but it might facilitate subcellular relocalization and interaction with additional partners [53]. Another potential key interactor of the extreme C-terminus of p73α (551–636 aa) is the receptor for activated C kinase-1 (RACK1). RACK1 overexpression indeed inhibits p73α-meditated transcription and p73α-dependent apoptosis. Deletion of the C-terminal region of p73α enhances its transactivation ability, suggesting that the C-terminal region of p73α exhibits a negative regulatory ability that modulates its transcriptional activity [26]. RACK1-mediated inhibition of p73α suggests the possibility that RACK1 acts as an adapter molecule between the transcriptional repressor and the C-terminal regulatory region of p73α. In addition, the interaction of RACK1 with the C-terminal region of p73α might alter the ability of p73α to interact with cellular proteins that have coactivator functions. RACK1 is currently the only player accounting for the differential transactivation potential of TAp73α and TAp73β.

## Concluding remarks and future perspective

Our current understanding indicates that various products from *TP73* genes, originated by alternative gene promoter usage and differential splicing, might differentially participate in complex networks that regulate cell growth, differentiation and death. However, still little evidence indicates proper selective mechanisms of action and pattern of expression of the individual C-terminal isoforms. It is important to investigate these isoforms to evaluate their contribution to physiology and identify their potential roles in human pathologies, such as cancer.

As current data indicate TAp73α as the exclusive, or major, expressed isoform in most of the biological system analyzed, a detailed spatio-temporal analysis of all the C-terminal isoforms expression pattern during development and tumorigenesis is critical to support a biological relevance for alternative splicings. To explore specificity of interaction partners for different C-terminal p73 isoforms and their unknown functions, it is also necessary to develop sensitive isoform-specific antibodies. The N-terminal isoform-specific KO mice shed considerable light onto the understanding of the roles and functions of these isoforms [10], [11], [19]. Thus, C-terminal isoform-specific KO mice models would be critical to unveil their functions and the potential correlations with human diseases. Previously uncovered common and unique features of C-terminal isoforms of p73 represent challenges to explore specific activities and regulation of each p73 isoform.

## References

[1] Kaghad M., Bonnet H., Yang A., Creancier L., Biscan J.C., Valent A., Minty A., Chalon P., Lelias J.M., Dumont X., Ferrara P., McKeon F., Caput D. (1997). Monoallelically expressed gene related to p53 at 1p36, a region frequently deleted in neuroblastoma and other human cancers. Cell.

[2] Yang A., Kaghad M., Wang Y., Gillett E., Fleming M.D., Dötsch V., Andrews N.C., Caput D., McKeon F. (1998). p63, a p53 homolog at 3q27–29, encodes multiple products with transactivating, death-inducing, and dominant-negative activities. Mol. Cell.

[3] De Laurenzi V., Raschellá G., Barcaroli D., Annicchiarico-Petruzzelli M., Ranalli M., Catani M.V., Tanno B., Costanzo A., Levrero M., Melino G. (2000). Induction of neuronal differentiation by p73 in a neuroblastoma cell line. J. Biol. Chem..

[4] Chillemi G., Kehrloesser S., Bernassola F., Desideri A., Dötsch V., Levine A.J., Melino G. (2017). Structural evolution and dynamics of the p53 proteins. Cold Spring Harb. Perspect. Med..

[5] Levine A.J., Tomasini R., McKeon F.D., Mak T.W., Melino G. (2011). The p53 family: guardians of maternal reproduction. Nat. Rev. Mol. Cell Biol..

[6] Senoo M., Pinto F., Crum C.P., McKeon F. (2007). p63 is essential for the proliferative potential of stem cells in stratified epithelia. Cell.

[7] Yang A., Schweitzer R., Sun D., Kaghad M., Walker N., Bronson R.T., Tabin C., Sharpe A., Caput D., Crum C., McKeon F. (1999). p63 is essential for regenerative proliferation in limb, craniofacial and epithelial development. Nature.

[8] Candi E., Amelio I., Agostini M., Melino G. (2015). MicroRNAs and p63 in epithelial stemness. Cell Death Differ..

[9] Lena A.M., Cipollone R., Amelio I., Catani M.V., Ramadan S., Browne G., Melino G., Candi E. (2010). Skn-1a/Oct-11 and ΔNp63α exert antagonizing effects on human keratin expression. Biochem. Biophys. Res. Commun..

[10] Tomasini R., Tsuchihara K., Wilhelm M., Fujitani M., Rufini A., Cheung C.C., Khan F., Itie-Youten A., Wakeham A., Tsao M.-S., Iovanna J.L., Squire J., Jurisica I., Kaplan D., Melino G., Jurisicova A., Mak T.W. (2008). TAp73 knockout shows genomic instability with infertility and tumor suppressor functions. Genes Dev..

[11] Yang A., Walker N., Bronson R., Kaghad M., Oosterwegel M., Bonnin J., Vagner C., Bonnet H., Dikkes P., Sharpe A., McKeon F., Caput D. (2000). p73-deficient mice have neurological, pheromonal and inflammatory defects but lack spontaneous tumours. Nature.

[12] Nemajerova A., Kramer D., Siller S.S., Herr C., Shomroni O., Pena T., Gallinas Suazo C., Glaser K., Wildung M., Steffen H., Sriraman A., Oberle F., Wienken M., Hennion M., Vidal R., Royen B., Alevra M., Schild D., Bals R., Dönitz J., Riedel D., Bonn S., Takemaru K.-I., Moll U.M., Lizé M. (2016). TAp73 is a central transcriptional regulator of airway multiciliogenesis. Genes Dev..

[13] Marshall C.B., Mays D.J., Beeler J.S., Rosenbluth J.M., Boyd K.L., Santos Guasch G.L., Shaver T.M., Tang L.J., Liu Q., Shyr Y., Venters B.J., Magnuson M.A., Pietenpol J.A. (2016). p73 is required for multiciliogenesis and regulates the Foxj1-associated gene network. Cell Rep..

[14] Nemajerova A., Amelio I., Gebel J., Dötsch V., Melino G., Moll U.M. (2018). Non-oncogenic roles of TAp73: from multiciliogenesis to metabolism. Cell Death Differ..

[15] Rinne T., Hamel B., van Bokhoven H., Brunner H.G. (2006). Pattern of p63 mutations and their phenotypes—update. Am. J. Med. Genet. A.

[16] Suzuki D., Sahu R., Leu N.A., Senoo M. (2015). The carboxy-terminus of p63 links cell cycle control and the proliferative potential of epidermal progenitor cells. Development.

[17] Rufini S., Lena A.M., Cadot B., Mele S., Amelio I., Terrinoni A., Desideri A., Melino G., Candi E. (2011). The sterile alpha-motif (SAM) domain of p63 binds in vitro monoasialoganglioside (GM1) micelles. Biochem. Pharmacol..

[18] Murray-Zmijewski F., Lane D.P., Bourdon J.-C. (2006). p53/p63/p73 isoforms: an orchestra of isoforms to harmonise cell differentiation and response to stress. Cell Death Differ..

[19] Wilhelm M.T., Rufini A., Wetzel M.K., Tsuchihara K., Inoue S., Tomasini R., Itie-Youten A., Wakeham A., Arsenian-Henriksson M., Melino G., Kaplan D.R., Miller F.D., Mak T.W. (2010). Isoform-specific p73 knockout mice reveal a novel role for delta Np73 in the DNA damage response pathway. Genes Dev..

[20] Melino G., De Laurenzi V., Vousden K.H. (2002). p73: friend or foe in tumorigenesis. Nat. Rev. Cancer.

[21] De Laurenzi V., Costanzo A., Barcaroli D., Terrinoni A., Falco M., Annicchiarico-Petruzzelli M., Levrero M., Melino G. (1998). Two new p73 splice variants, gamma and delta, with different transcriptional activity. J. Exp. Med..

[22] Thanos C.D., Bowie J.U. (1999). p53 family members p63 and p73 are SAM domain-containing proteins. Protein Sci..

[23] Chi S.-W., Ayed A., Arrowsmith C.H. (1999). Solution structure of a conserved C-terminal domain of p73 with structural homology to the SAM domain. EMBO J..

[24] Arrowsmith C.H. (1999). Structure and function in the p53 family. Cell Death Differ..

[25] Liu G., Chen X. (2005). The C-terminal sterile alpha motif and the extreme C terminus regulate the transcriptional activity of the alpha isoform of p73. J. Biol. Chem..

[26] Ueda Y., Hijikata M., Takagi S., Chiba T., Shimotohno K. (1999). New p73 variants with altered C-terminal structures have varied transcriptional activities. Oncogene.

[27] Ishimoto O., Kawahara C., Enjo K., Obinata M., Nukiwa T., Ikawa S. (2002). Possible oncogenic potential of DeltaNp73: a newly identified isoform of human p73. Cancer Res..

[28] Ozaki T., Naka M., Takada N., Tada M., Sakiyama S., Nakagawara A. (1999). Deletion of the COOH-terminal region of p73alpha enhances both its transactivation function and DNA-binding activity but inhibits induction of apoptosis in mammalian cells. Cancer Res..

[29] Davison T.S., Vagner C., Kaghad M., Ayed A., Caput D., Arrowsmith C.H. (1999). p73 and p63 are homotetramers capable of weak heterotypic interactions with each other but not with p53. J. Biol. Chem..

[30] Amelio I., Melino G. (2015). The p53 family and the hypoxia-inducible factors (HIFs): determinants of cancer progression. Trends Biochem. Sci..

[31] Der Ou H., Löhr F., Vogel V., Mäntele W., Dötsch V. (2007). Structural evolution of C-terminal domains in the p53 family. EMBO J..

[32] Grespi F., Amelio I., Tucci P., Annicchiarico-Petruzzelli M., Melino G. (2012). Tissue-specific expression of p73 C-terminal isoforms in mice. Cell Cycle.

[33] Rufini A., Agostini M., Grespi F., Tomasini R., Sayan B.S., Niklison-Chirou M.V., Conforti F., Velletri T., Mastino A., Mak T.W., Melino G., Knight R.A. (2011). p73 in cancer. Genes Cancer.

[34] Zaika A.I., Kovalev S., Marchenko N.D., Moll U.M. (1999). Overexpression of the wild type p73 gene in breast Cancer tissues and cell lines. Cancer Res..

[35] Gonzalez S., Perez-Perez M.M., Hernando E., Serrano M., Cordon-Cardo C. (2005). p73β-mediated apoptosis requires p57^kip2^ induction and IEX-1 inhibition. Cancer Res..

[36] Tomasini R., Mak T.W., Melino G. (2008). The impact of p53 and p73 on aneuploidy and cancer. Trends Cell Biol..

[37] Vernole P., Neale M.H., Barcaroli D., Munarriz E., Knight R.A., Tomasini R., Mak T.W., Melino G., De Laurenzi V. (2009). TAp73α binds the kinetochore proteins Bub1 and Bub3 resulting in polyploidy. Cell Cycle.

[38] Amelio I., Inoue S., Markert E.K., Levine A.J., Knight R.A., Mak T.W., Melino G. (2015). TAp73 opposes tumor angiogenesis by promoting hypoxia-inducible factor 1α degradation. Proc. Natl. Acad. Sci. U. S. A..

[39] Stantic M., Sakil H.A.M., Zirath H., Fang T., Sanz G., Fernandez-Woodbridge A., Marin A., Susanto E., Mak T.W., Arsenian Henriksson M., Wilhelm M.T. (2015). TAp73 suppresses tumor angiogenesis through repression of proangiogenic cytokines and HIF-1α activity. Proc. Natl. Acad. Sci..

[40] Amelio I., Markert E.K., Rufini A., Antonov A.V., Sayan B.S., Tucci P., Agostini M., Mineo T.C., Levine A.J., Melino G. (2014). p73 regulates serine biosynthesis in cancer. Oncogene.

[41] Velletri T., Romeo F., Tucci P., Peschiaroli A., Annicchiarico-Petruzzelli M., Niklison-Chirou M.V., Amelio I., Knight R.A., Mak T.W., Melino G., Agostini M. (2013). GLS2 is transcriptionally regulated by p73 and contributes to neuronal differentiation. Cell Cycle.

[42] Du W., Jiang P., Mancuso A., Stonestrom A., Brewer M.D., Minn A.J., Mak T.W., Wu M., Yang X. (2013). TAp73 enhances the pentose phosphate pathway and supports cell proliferation. Nat. Cell Biol..

[43] Niklison-Chirou M.V., Erngren I., Engskog M., Haglöf J., Picard D., Remke M., McPolin P.H.R., Selby M., Williamson D., Clifford S.C., Michod D., Hadjiandreou M., Arvidsson T., Pettersson C., Melino G., Marino S. (2017). TAp73 is a marker of glutamine addiction in medulloblastoma. Genes Dev..

[44] Gurpinar E., Vousden K.H. (2015). Hitting cancers' weak spots: vulnerabilities imposed by p53 mutation. Trends Cell Biol..

[45] Freed-Pastor W.A., Prives C. (2012). Mutant p53: one name, many proteins. Genes Dev..

[46] Kehrloesser S., Osterburg C., Tuppi M., Schäfer B., Vousden K.H., Dötsch V. (2016). Intrinsic aggregation propensity of the p63 and p73 TI domains correlates with p53R175H interaction and suggests further significance of aggregation events in the p53 family. Cell Death Differ..

[47] Kruse J.-P., Gu W. (2009). Modes of p53 regulation. Cell.

[48] Hosoda M., Ozaki T., Miyazaki K., Hayashi S., Furuya K., Watanabe K., Nakagawa T., Hanamoto T., Todo S., Nakagawara A. (2005). UFD2a mediates the proteasomal turnover of p73 without promoting p73 ubiquitination. Oncogene.

[49] Rossi M., De Laurenzi V., Munarriz E., Green D.R., Liu Y.-C., Vousden K.H., Cesareni G., Melino G. (2005). The ubiquitin-protein ligase itch regulates p73 stability. EMBO J..

[50] Sudol M. (1996). Structure and function of the WW domain. Prog. Biophys. Mol. Biol..

[51] Miyazaki K., Ozaki T., Kato C., Hanamoto T., Fujita T., Irino S., Watanabe K., Nakagawa T., Nakagawara A. (2003). A novel HECT-type E3 ubiquitin ligase, NEDL2, stabilizes p73 and enhances its transcriptional activity. Biochem. Biophys. Res. Commun..

[52] Asher G., Tsvetkov P., Kahana C., Shaul Y. (2005). A mechanism of ubiquitin-independent proteasomal degradation of the tumor suppressors p53 and p73. Genes Dev..

[53] Minty A., Dumont X., Kaghad M., Caput D. (2000). Covalent modification of p73 by SUMO-1: two-hybrid screening with p73 identifies novel SUMO-1-interacting proteins and a SUMO-1 interaction motif. J. Biol. Chem..

